# The Originator of the Quinine Industry

**Published:** 1896-03

**Authors:** 


### The Originator of the Quinine Industry.

The *Agricultural Gazette*, of New South Wales, states that there is still living at Kenmore, in excellent health, Mr. Charles Ledger, the man, who, forty years ago, after most perilous adventures, introduced the variety of cinchona calisaya known as ledgeriana into the island of Java. Messrs. Howard & Sons, the great quinine firm, say that the supply of Peruvian bark from Java is almost all from the ledgeriana trees, the only complaint against this variety being that it has turned out so rich that the trees are supplying too much quinine for the world to consume. Perhaps the quantity of bark which is now produced every year from seed furnished by Mr. Ledger cannot be short of ten million pounds, and to him, more than any one else, perhaps, is due the fact that quinine has been brought within the means of the very poorest.

